# Characterisation of early responses in lead accumulation and localization of *Salix babylonica* L. roots

**DOI:** 10.1186/s12870-020-02500-6

**Published:** 2020-06-29

**Authors:** Wenxiu Xue, Yi Jiang, Xiaoshuo Shang, Jinhua Zou

**Affiliations:** Tianjin Key Laboratory of Animal and Plant Resistance, College of Life Science, Tianjin Normal University, Tianjin, 300387 China

**Keywords:** Energy-dispersive X-ray analyses (EDXA), Fluorescence labeling, Lead (Pb), Propidium iodide (PI), *Salix babylonica* L., Subcellular localization

## Abstract

**Background:**

Lead (Pb) is a harmful pollutant that disrupts normal functions from the cell to organ levels. *Salix babylonica* is characterized by high biomass productivity, high transpiration rates, and species specific Pb. Better understanding the accumulating and transporting Pb capability in shoots and roots of *S. babylonica*, the toxic effects of Pb and the subcellular distribution of Pb is very important.

**Results:**

Pb exerted inhibitory effects on the roots and shoots growth at all Pb concentrations. According to the results utilizing inductively coupled plasma atomic emission spectrometry (ICP-AES), *S. babylonica* can be considered as a plant with great phytoextraction potentials as translocation factor (TF) value > 1 is observed in all treatment groups throughout the experiment. The Leadmium™ Green AM dye test results indicated that Pb ions initially entered elongation zone cells and accumulated in this area. Then, ions were gradually accumulated in the meristem zone. After 24 h of Pb exposure, Pb accumulated in the meristem zone. The scanning electron microscopy (SEM) and energy-dispersive X-ray analyses (EDXA) results confirmed the fluorescent probe observations and indicated that Pb was localized to the cell wall and cytoplasm. In transverse sections of the mature zone, Pb levels in the cell wall and cytoplasm of epidermal cells was the lowest compared to cortical and vessel cells, and an increasing trend in Pb content was detected in cortical cells from the epidermis to vascular cylinder. Similar results were shown in the Pb content in the cell wall and cytoplasm of the transverse sections of the meristem. Cell damage in the roots exposed to Pb was detected by propidium iodide (PI) staining, which was in agreement with the findings of Pb absorption in different zones of *S. babylonica* roots under Pb stress.

**Conclusion:**

*S. babylonica* L. is observed as a plant with great potential of Pb-accumulation and Pb-tolerance. The information obtained here of Pb accumulation and localization in *S. babylonica* roots can furthers our understanding of Pb-induced toxicity and its tolerance mechanisms, which will provide valuable and scientific information to phytoremediation investigations of other woody plants under Pb stress.

## Background

Nowadays, the rapid growth of industrialization and human activities, such as mining and smelting of lead (Pb) ores, can result in environmental Pb pollution and its entering into the food chain, which poses a great risk to the health of both plants and human beings [1, 2]. Therefore, Pb pollution has been becoming a critical social problem today [3]. Among all the heavy metals, Pb toxicity is only lower than As [4]. It is not only non-essential for plant growth, but often toxic to plant metabolism. Pb disrupts normal functions from the cell to organ levels, such as Pb inducing damage of root tip meristematic cells and guard cells in leaves and disturbing the function of chloroplast, mitochondria, nucleolus and vacuole [5–8]. Evidence has demonstrated that Pb can be easily absorbed, transformed, and accumulated in plant tissues, in which the roots are the primary sites of accumulation [9–12]. A higher percentage of accumulated Pb is restricted within the roots, while only a small fraction of it is transported to the aerial parts of plants [13, 14]. Previous reports demonstrated that Pb toxicity is associated with the inducement of a low mitotic index, disturbance of mitosis, visible chloroplast alterations, plant cell malformations, mitochondrial system abnormalities, inward invagination of cell walls, plasma membrane distortions, oversized vacuoles, and irregular plastoglobuli formations [15–21].

The *Salicaceae* family contains the *Salix* (willows) and *Populus* (poplars) genera, which are comprised of several woody species and hybrids. Many of them have accommodated to certain ecological niches, for example the places that are nutrient-poor, dry, wet or contaminated by metal [22, 23]. Willows with the characteristics of high biomass, accumulating and translocating heavy metals to shoots easily are considered as excellent phytoremediation species [24–27]. *S. babylonica* is a willow species that grows in a wide range of climatic conditions and is one of the most widely cultivated willow species in China [28]. This species is preferred for the phytoremediation of trace metal-contaminated land due to its easy propagation and cultivation, fast growth, large biomass, and deep root system [23, 28]. An early study reported that *S. babylonica* tolerates and accumulates Pb, suggesting that it has considerable potential for remediating Pb pollution [29]. However, little is known in this respect.

Phytoremediation is an economic way which utilize the potential of plants to transform or eliminate the environmental contaminants by accumulating in their tissues, which is a cheap alternative that complements common, conventional methods [30, 31]. The success of phytoextraction is mainly determined by the identification of native high biomass yielding, the capability of the plants for the heavy metal accumulation and translocation, the tolerance to high heavy metal concentrations [32]. Better understanding the uptake, accumulation, transportation and distribution of heavy metals in plants and the toxic effects on tissues, organs and cells are needed and very important.

In the present investigation, the early responses of *S. babylonica* roots exposed to different Pb concentrations in respect to Pb uptake and accumulation, subcellular distribution, and the toxic effects of Pb on plant growth and cell damage were studied by utilizing inductively coupled plasma atomic emission spectrometry (ICP-AES), fluorescence labeling, propidium iodide (PI) staining, scanning electron microscopy (SEM), and energy-dispersive X-ray analyses (EDXA). The data will be very valuable in better our understanding of Pb-induced toxicity and the associated tolerance mechanisms in woody plants under Pb stress.

## Results

### Effects of Pb on seedling growth

**Fig. 1:**
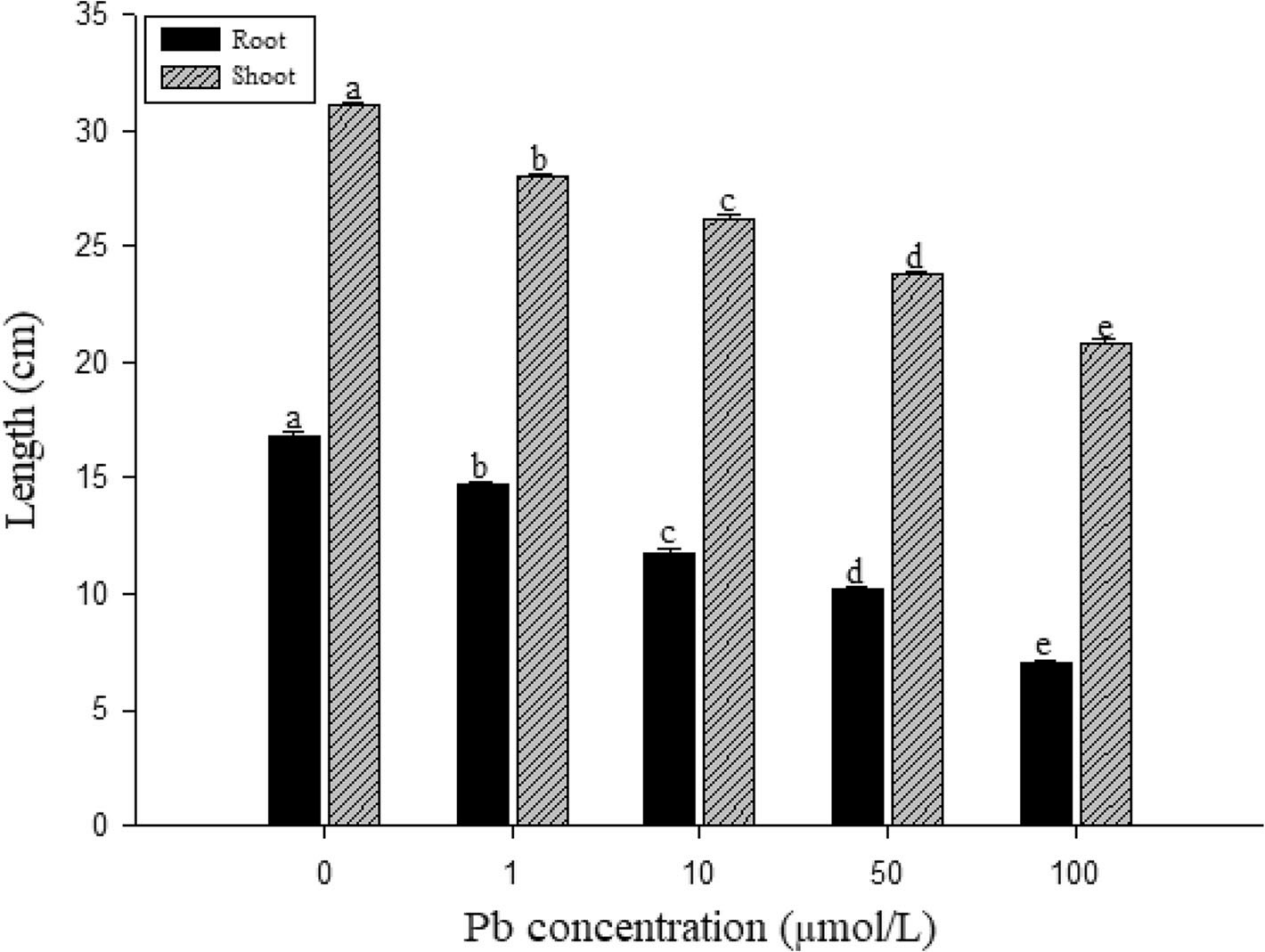
Effects of Pb on *S. babylonica* root and shoot length exposed to 0, 1, 10, 50, or 100 μmol/L Pb for 7 d. Vertical bars denote the SE. Different letters indicate significant differences (*n* = 10, *p* < 0.05)

The Pb effects on *S. babylonica* root growth varied with different Pb concentrations after 7 d (Fig. 1). Compared to the control, Pb exerted significant inhibitory effects on roots and shoots (*p* < 0.05). The data also revealed that both root and shoot length decreased significantly (*p* < 0.05) as Pb concentrations increased.

### Pb accumulation

The ICP-AES data showed that the Pb levels in *S. babylonica* roots exposed to Pb solution for 7 d increased significantly (*p* < 0.05) when compared to the control and exhibited a gradually increasing trend as Pb concentrations increased (1, 10, 50 and 100 μmol/L) (Table 1). The Pb content in the stems and leaves exhibited the same trend as the roots. Pb accumulation in shoots was higher than that in the roots. At 100 μmol/L Pb treatment for 7 d, the root Pb accumulation was 78.78 ± 0.34 μg/g dry weight of the tissue, and the shoot Pb accumulation was 151.37 ± 0.16 μg/g dry weight of the tissue.

**Table 1:** Pb levels of different *S. babylonica* organs exposed to different Pb concentrations for 7 d

Treatment (μmol/L)	Dry weight (μg/g) ± SE			TF
	Stem	Leaf	Root	
0	9.91 ± 0.04a	8.50 ± 0.43a	11.50 ± 0.27a	1.60
1	41.50 ± 0.50b	33.51 ± 0.91b	31.56 ± 0.65b	2.38
10	70.85 ± 0.06c	45.79 ± 0.01c	48.91 ± 0.05c	2.38
50	83.08 ± 0.17d	49.48 ± 0.09d	59.43 ± 0.04d	2.23
100	98.38 ± 0.13e	52.99 ± 0.03e	78.78 ± 0.34e	1.92

Different letters indicate significant differences (*p* < 0.05). Data are presented as mean ± SE (*n* = 5)

Translocation factors (TF) of all treatments were also calculated, the data also confirmed the Pb accumulation in *S. babylonica* shoots and roots under Pb stress. The highest TF was found at 1 and 10 μmol/L Pb treatment, and there was a decreasing trend in the TF values as Pb concentrations increased (Table 1). However, the TF value > 1 was observed in all treatment groups throughout the experiment (Table 1).

### Effects of Pb on cell damage

**Fig. 2:**
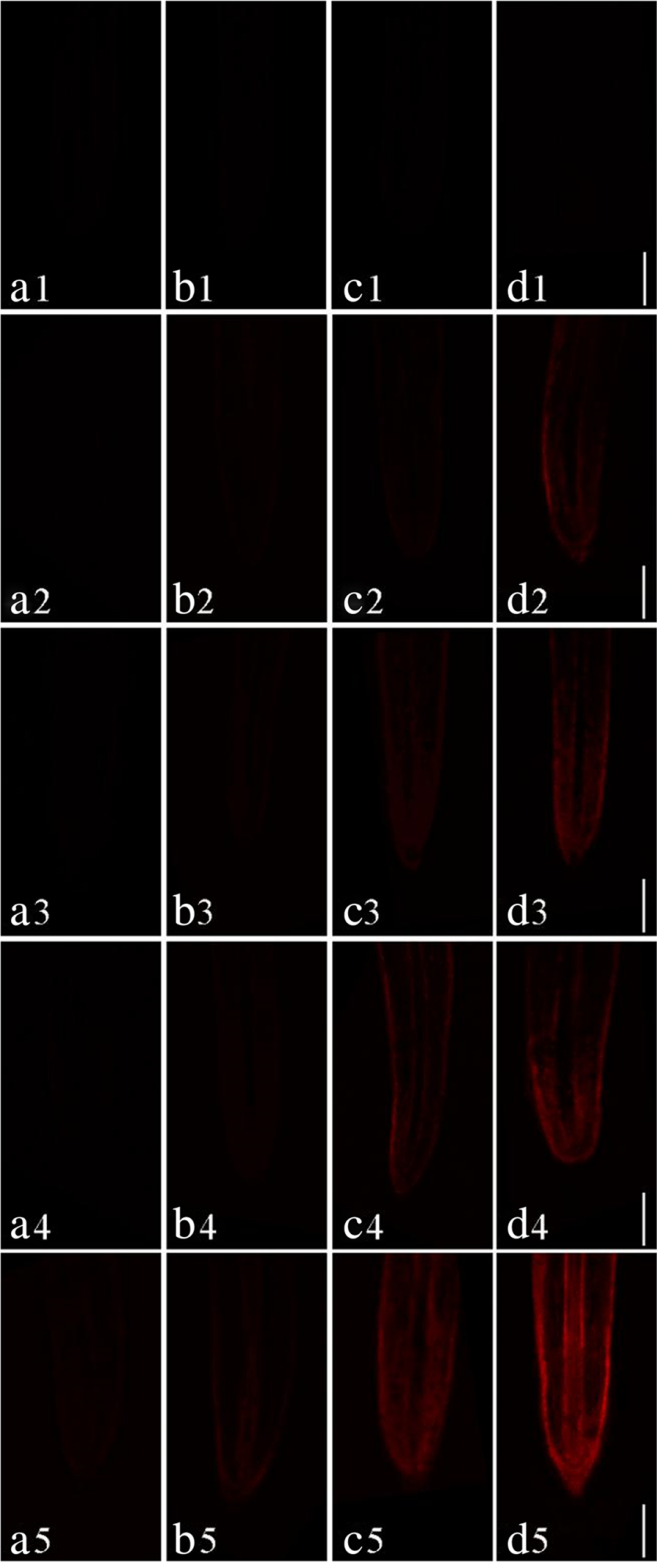
Micrographs of *S. babylonica* roots using PI dye at longitudinal root tips exposed to different Pb concentrations (0, 1, 10, 50, or 100 μmol/L) for different treatment times (0, 3, 6, 12, and 24 h). A1–D1: Control without Pb for 3, 6, 12, and 24 h; A2–D2: 1 μmol/L Pb for 3, 6, 12, and 24 h; A3–D3: 10 μmol/L Pb for 3, 6, 12, and 24 h; A4–D4: 50 μmol/L Pb for 3, 6, 12, and 24 h; A5–D5: 100 μmol/L Pb for 3, 6, 12, and 24 h. Scale bar = 1 mm

**Fig. 3:**
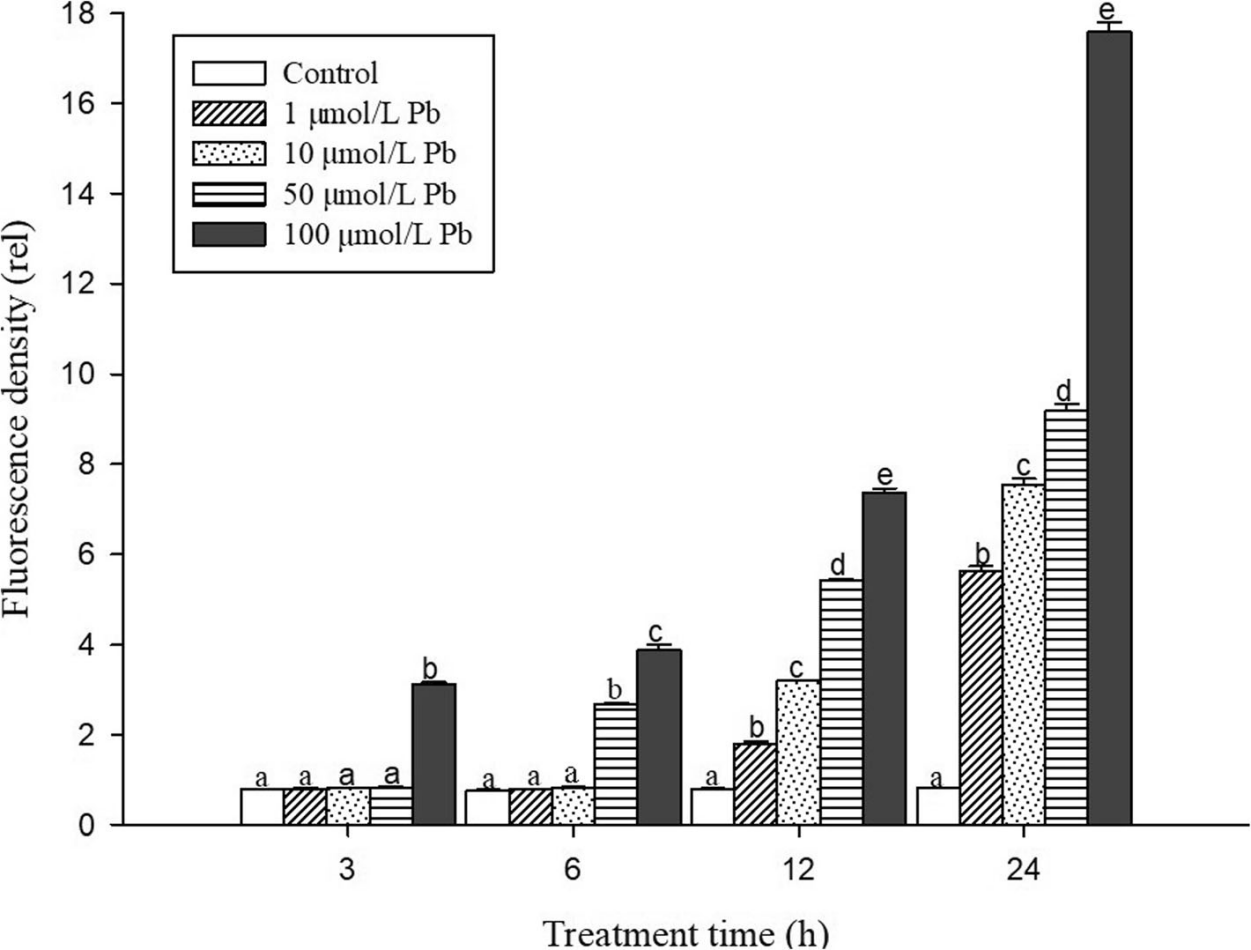
Analysis of PI fluorescence density detected by Image J at longitudinal sections of roots exposed to 1, 10, 50, or 100 μmol/L Pb for 3, 6, 12, and 24 h. Vertical bars denote the SE. Different letters indicate significant differences (*p* < 0.05)

**Fig. 4:**
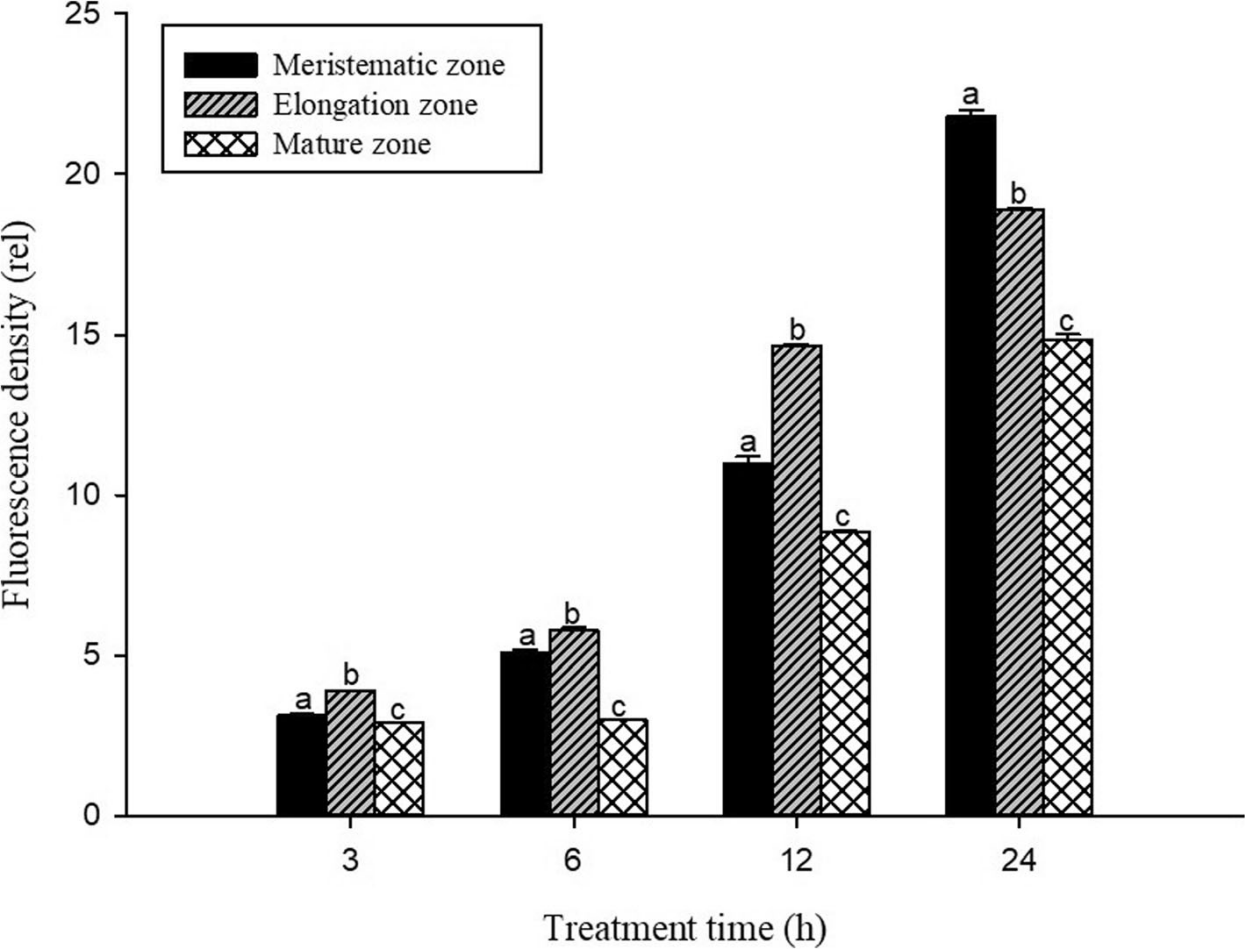
Distribution of PI fluorescence density detected by Image J in different zones of *S. babylonica* roots treated with 100 μmol/L Pb for 3, 6, 12, and 24 h. Vertical bars denote the SE. Different letters indicate significant differences (*p* < 0.05)

In order to investigate the toxic effects of Pb on cell damage in root tips, PI dyes were used to visualize dead cells at longitudinal sections of *S. babylonica* root tips exposed to 0, 1, 10, 50, or 100 μmol/L Pb for 3, 6, 12, and 24 h (Fig. 2a–d). Red fluorescence is an indicator of cell damage. In the root tips of *S. babylonica*, the different Pb concentrations at different treatment time were responsible for the degree of cell damage caused by Pb (Fig. 2a–d). A significant red fluorescence signal was not observed in the control roots (Fig. 2A1–D1). Weak red fluorescence labeling gradually appeared in root tip cells exposed to 1 or 10 μmol/L Pb for 12 h (Fig. 2C2, C3), 50 μmol/L Pb for 6 h (Fig. 2B4), and 100 μmol/L Pb for 3 h (Fig. 2A5), indicating that Pb could induce cell damage as soon as 3 h after Pb exposure. Fluorescence intensity was more pronounced as Pb concentrations increased and due to prolonged exposure. The strongest fluorescence was observed in root tip cells treated with 100 μmol/L Pb. Data from the fluorescence density analysis confirmed these findings (Fig. 3). Cell damage increased significantly as Pb concentrations and treatment time increased (*p* < 0.05). In *S. babylonica* root tips, the Pb accumulation and location and the distribution of cell death are almost the same. The results showed the distribution of cell damage in various root areas after *S. babylonica* was stressed by 100 μmol/L Pb for 3, 6, 12 and 24 h (Fig. 4). When compared with meristem and mature zones, the degree of necrotic cells in the elongation zone was greatly higher after 3–12 h of Pb exposure (*p* < 0.05). After exposure to 100 μmol/L Pb for 24 h, the level of necrotic cells in the meristem zone was significantly higher (*p* < 0.05) compared to the elongation and mature zones.

**Fig. 5:**
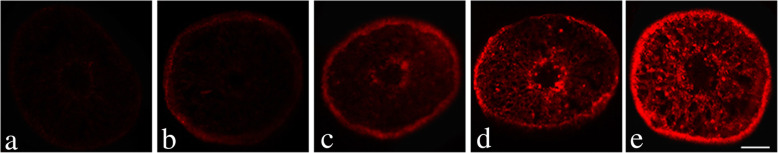
Micrographs of *S. babylonica* roots using PI dye at transverse sections of root mature zone exposed to different Pb concentrations (0, 1, 10, 50, or 100 μmol/L) for 24 h. Scale bar = 200 μm. **a**: Control; **b**: 1 μmol/L Pb; **c**: 10 μmol/L Pb; **d**: 50 μmol/L Pb; **e**: 100 μmol/L Pb

**Fig. 6:**
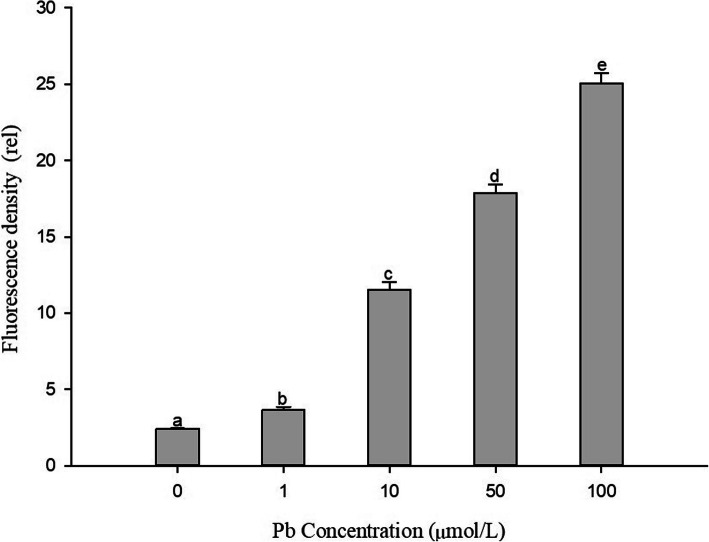
Analysis of PI fluorescence density at transverse sections of root mature zone exposed to 1, 10, 50, or 100 μmol/L Pb for 24 h. Vertical bars denote the SE. Different letters indicate significant differences (*p* < 0.05)

Cell damage was observed in transverse sections of the mature zone of *S. babylonica* roots exposed to 0, 1, 10, 50, or 100 μmol/L Pb for 24 h (Fig. 5a–e). In control root tip cells, there was very weak red fluorescence been observed, indicating a little cell damage in the normal plant growth (Fig. 5a). After 24 h in 1 μmol/L Pb treatment, compared to the control, stronger red fluorescence was detected suggesting more dead cells were induced by Pb in *S. babylonica* roots (Fig. 5b). This toxic effect increased as Pb concentrations increased (Fig. 5c–e). At low Pb concentrations (1 μmol/L), low levels of fluorescence intensity were mainly concentrated in epidermal and cortical cells near the epidermis (Fig. 5b). As Pb concentrations increased, strong red fluorescence was observed. The toxic effects of Pb on cell damage were observed in cortical cells near the vascular column of roots exposed to 10 μmol/L Pb (Fig. 5C). Increasing fluorescence intensity appeared in all root cortical cells treated with 50 or 100 μmol/L Pb (Fig. 5d, e). The fluorescence density analysis revealed significant Pb-induced cell damage at transverse sections of *S. babylonica* roots under Pb stress (*p* < 0.05); this toxic effect increased as Pb concentrations increased (Fig. 6).

### Pb distribution in root tips

**Fig. 7:**
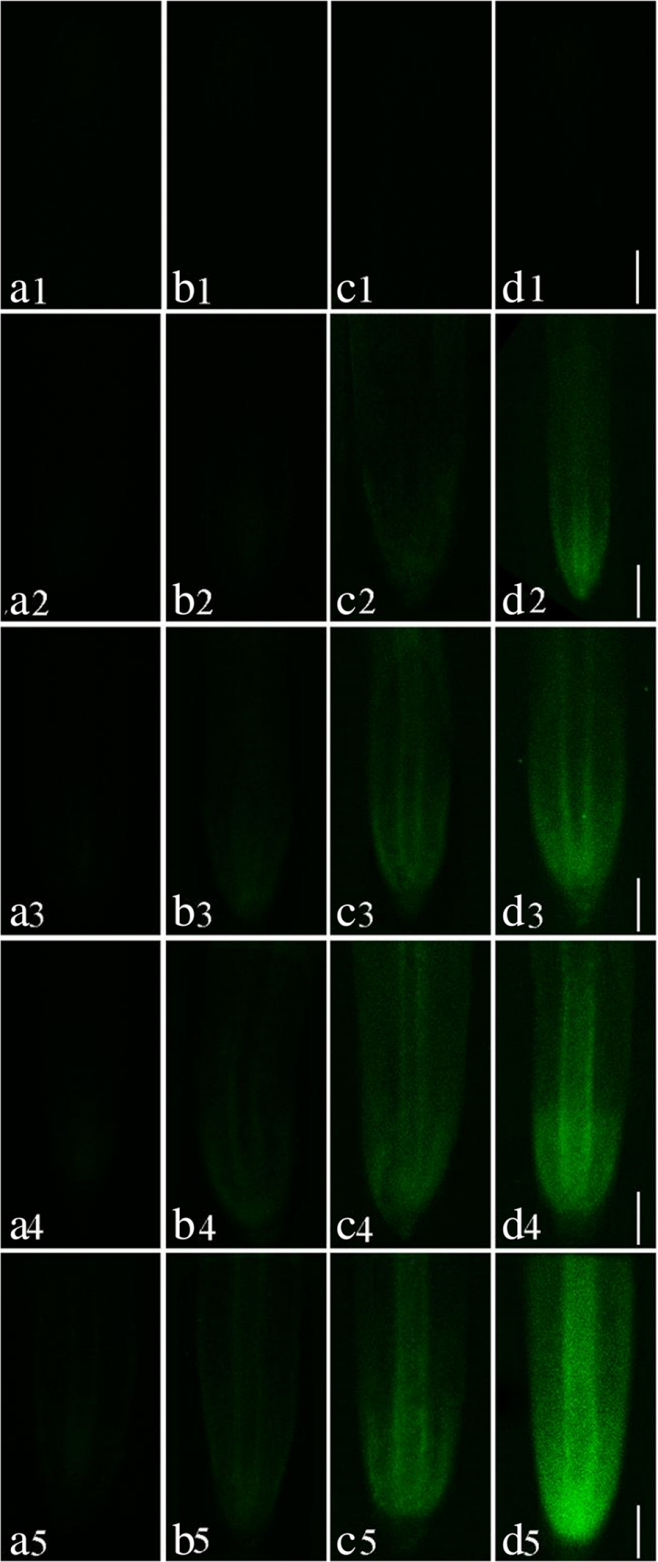
Micrographs of *S. babylonica* roots using Leadmium Green AM dye at longitudinal sections of roots exposed to different Pb concentrations (0, 1, 10, 50, or 100 μmol/L) for different treatment times (3, 6, 12, and 24 h). a1–d1: Control without Pb for 3, 6, 12, and 24 h; a2–d2: 1 μmol/L Pb for 3, 6, 12, and 24 h; a3–d3: 10 μmol/L Pb for 3, 6, 12, and 24 h; a4–d4: 50 μmol/L Pb for 3, 6, 12, and 24 h; a5–d5: 100 μmol/L Pb for 3, 6, 12, and 24 h. Scale bar = 1 mm

**Fig. 8:**
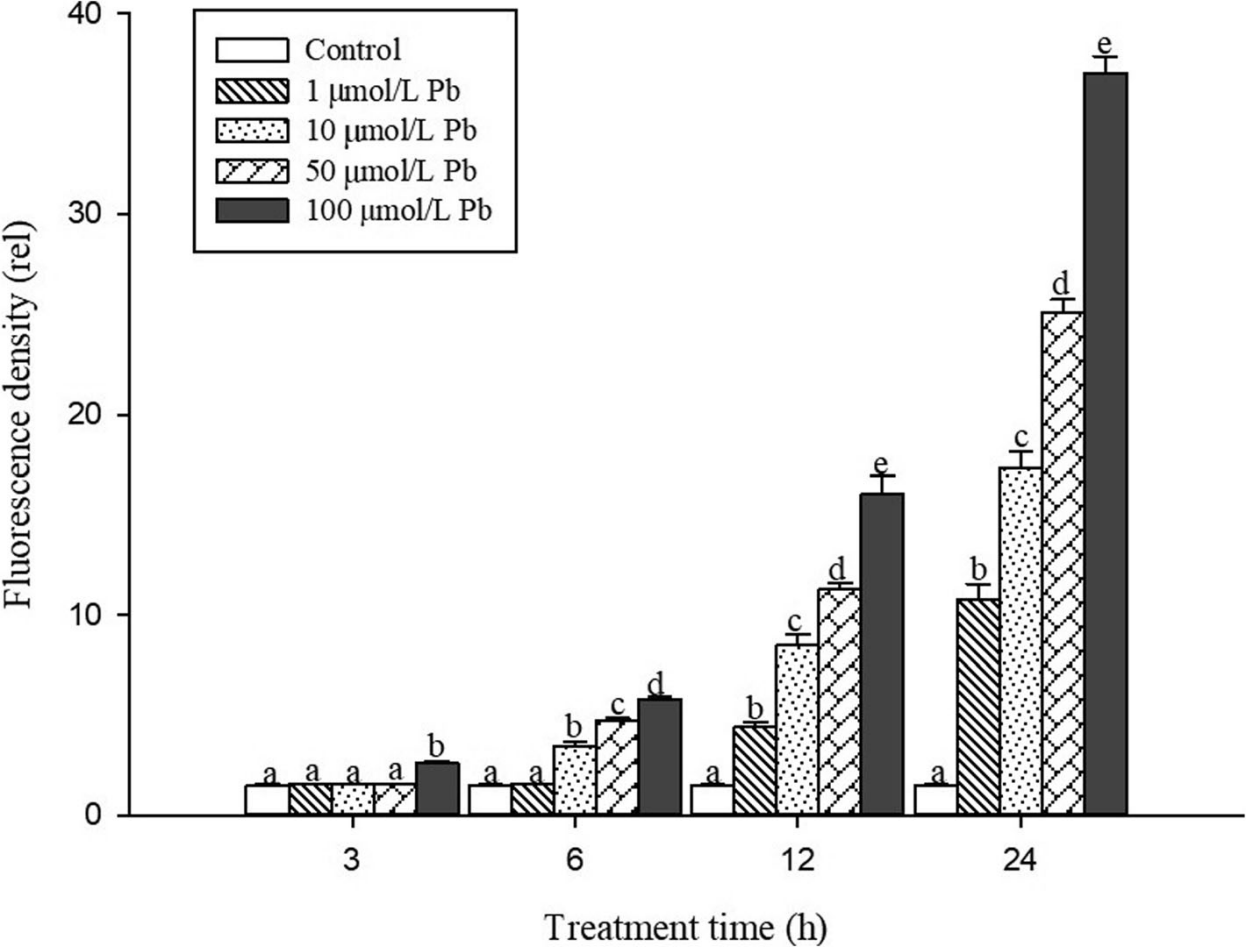
Analysis of Leadamium™ Green AM dye fluorescence density detected by Image J at longitudinal sections of roots pretreated with 0, 1, 10, 50, or 100 μmol/L Pb for 3, 6, 12, and 24 h. Vertical bars denote the SE. Different letters indicate significant differences (*p* < 0.05)

**Fig. 9:**
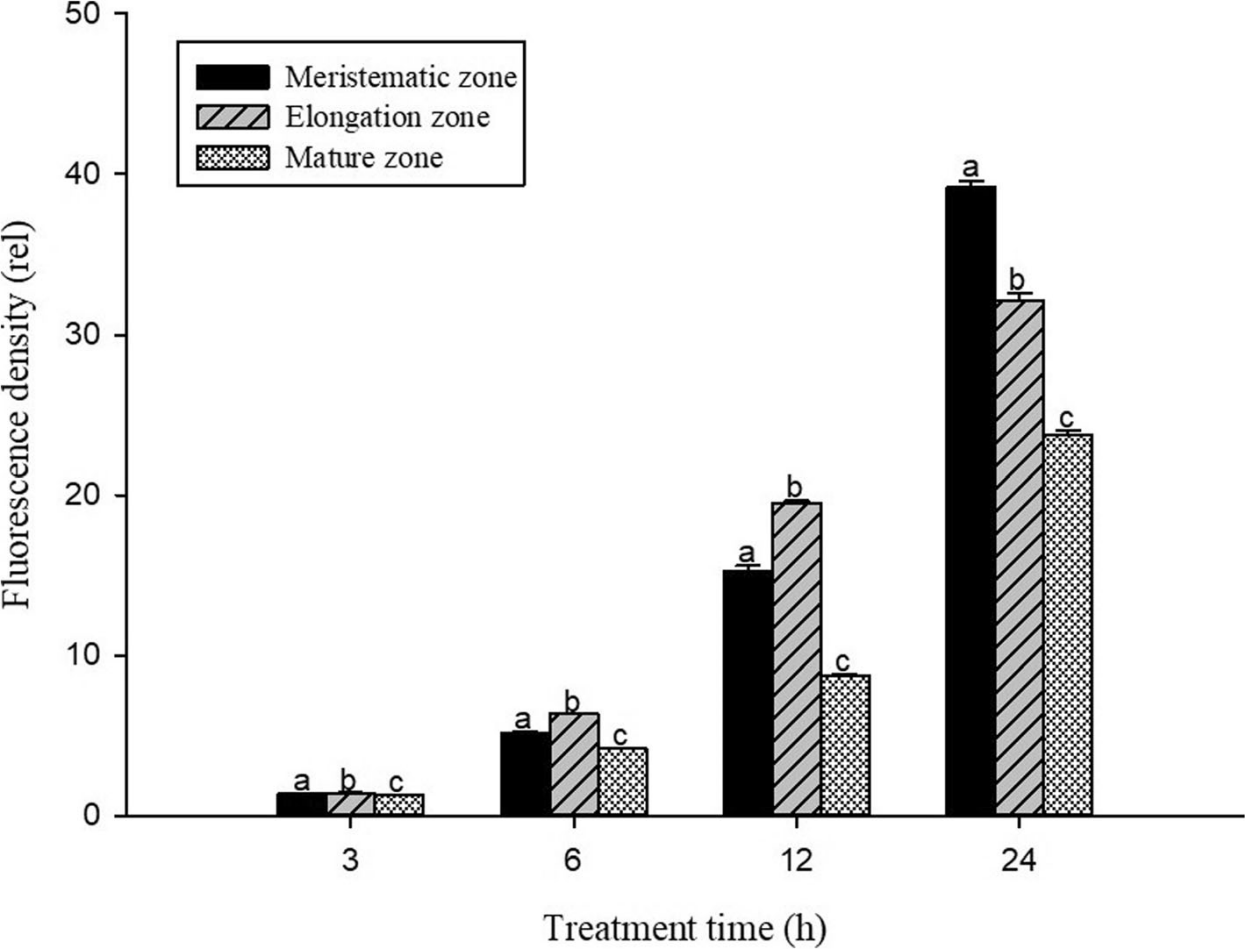
Distribution of Leadamium™ Green AM dye fluorescence density in the meristem, elongation, and mature zones at longitudinal sections of root tips treated with 100 μmol/L Pb for 3, 6, 12, and 24 h

The Pb distribution in *S. babylonica* root tips exposed to 0, 1, 10, 50, or 100 μmol/L Pb for 3, 6, 12, and 24 h was conducted using a Pb-specific Leadmium Green AM dye probe. The fluorescent dye revealed a bright and clear green fluorescence in root tip cells of Pb-treated roots due to the Pb specific probe Leadmium™ Green AM solution (Fig. 7a–d). A significant green fluorescence signal was not detected in control root tips (Fig. 7A1–D1). A weak green fluorescence signal appeared first in the root tip cells exposed to 10, 50, or 100 μmol/L Pb for 3 h (Fig. 7A3–A5) compared to the control. This phenomenon was also observed in roots exposed to 1 μmol/L Pb for 6 h (Fig. 7B2). These data revealed that Pb could enter root cells after 3 h. The labeling of root tip cells increased as Pb concentrations increased and due to prolonged exposure. The strongest fluorescence in root tip cells was observed in 100 μmol/L Pb (Fig 7A5–D5). The fluorescence density analysis confirmed the above observations (Fig. 8). The Pb distribution in the 3 zones of *S. babylonica* roots treated with 100 μmol/L Pb for 3, 6, 12, and 24 h was analyzed by Image J (Fig. 9). The Pb levels in the 3 zones of roots exposed to Pb for 12 h were significantly different (*p* < 0.05) and ordered as follows: elongation zone > meristem zone > mature zone. The order of Pb contents after 24 h exposure was as follows: meristem area > elongation area > mature area. The above results showed that Pb absorbed and accumulated mainly in the meristem and elongation zones. These results exhibited the same trend in root cells damaged by Pb (Fig. 4).

### Subcellular localization of Pb

**Fig. 10:**
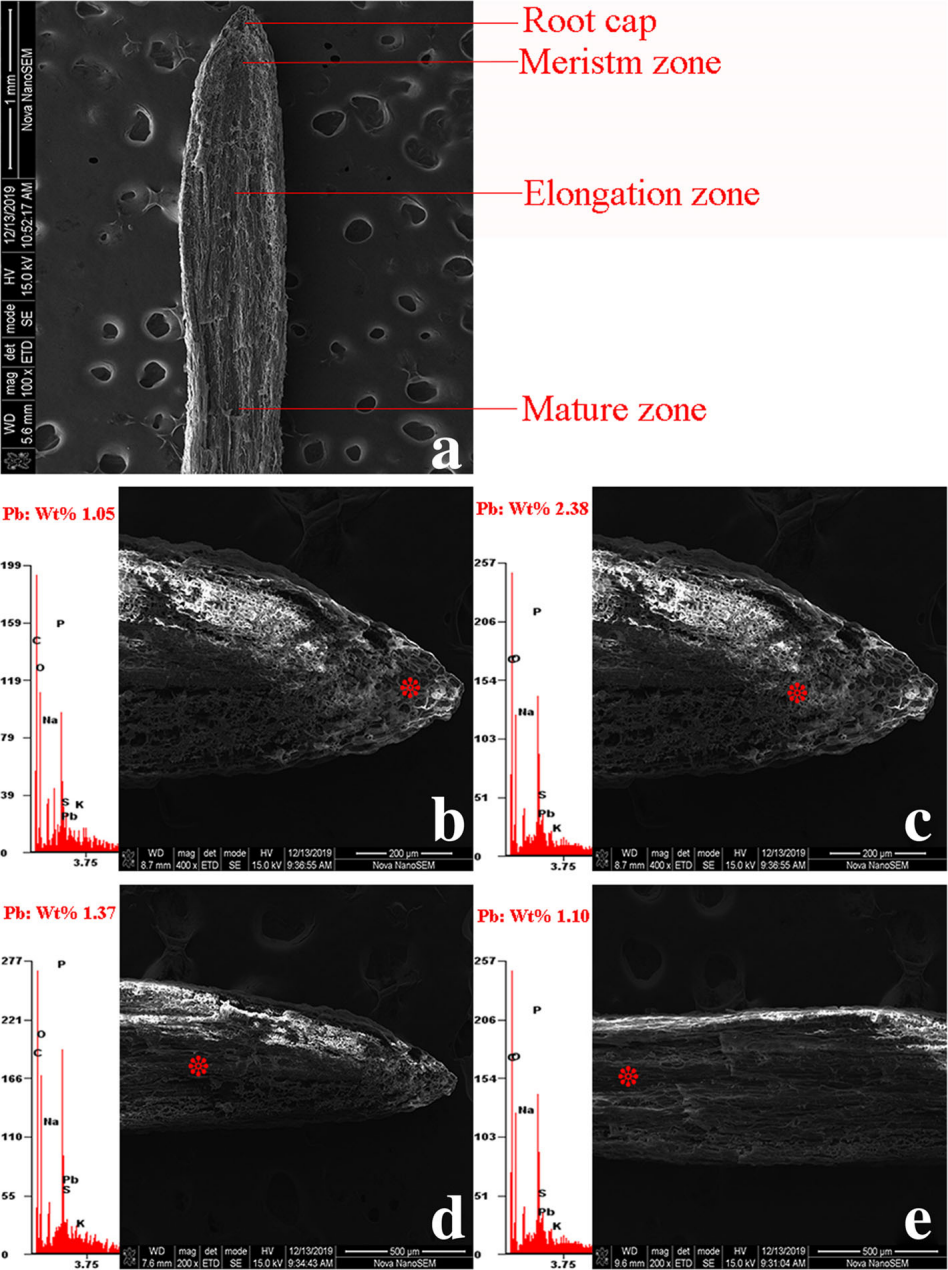
SEM micrographs and Pb localization in different zones of root tip cells exposed to 50 μmol/L Pb for 24 h. **a**: Intact root (scale bar = 1 mm), **b**: Root cap (scale bar = 200 μm), **c**: Meristem zone (scale bar = 200 μm), **d**: Elongation zone (scale bar = 500 μm), **e**: Mature zone (scale bar = 500 μm). site of the analysis; x-axis energy [keV]

**Fig. 11:**
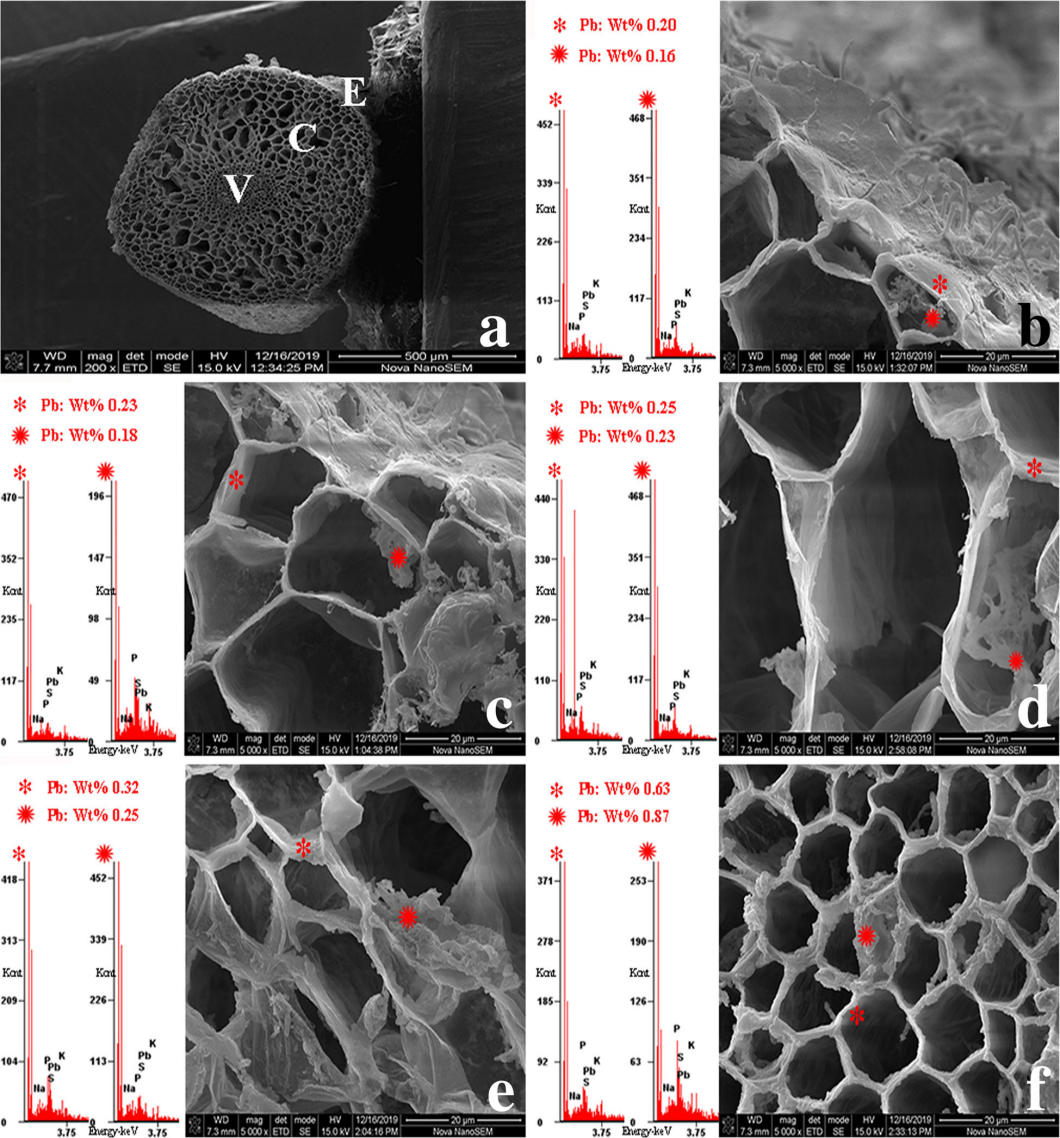
SEM micrographs and Pb localization at transverse sections in root mature zone cells exposed to 50 μmol/L Pb for 24 h. **a**: Transverse sections of the mature zone (scale bar = 500 μm), **b**: Epidermal cells (scale bar = 20 μm), **c**–**e**: Cortical cells (scale bar = 20 μm), **f**: Vessel cells (scale bar = 20 μm). *cell wall, cytoplasm, E: epidermis, C: cortex, and V: vascular cylinder

**Fig. 12:**
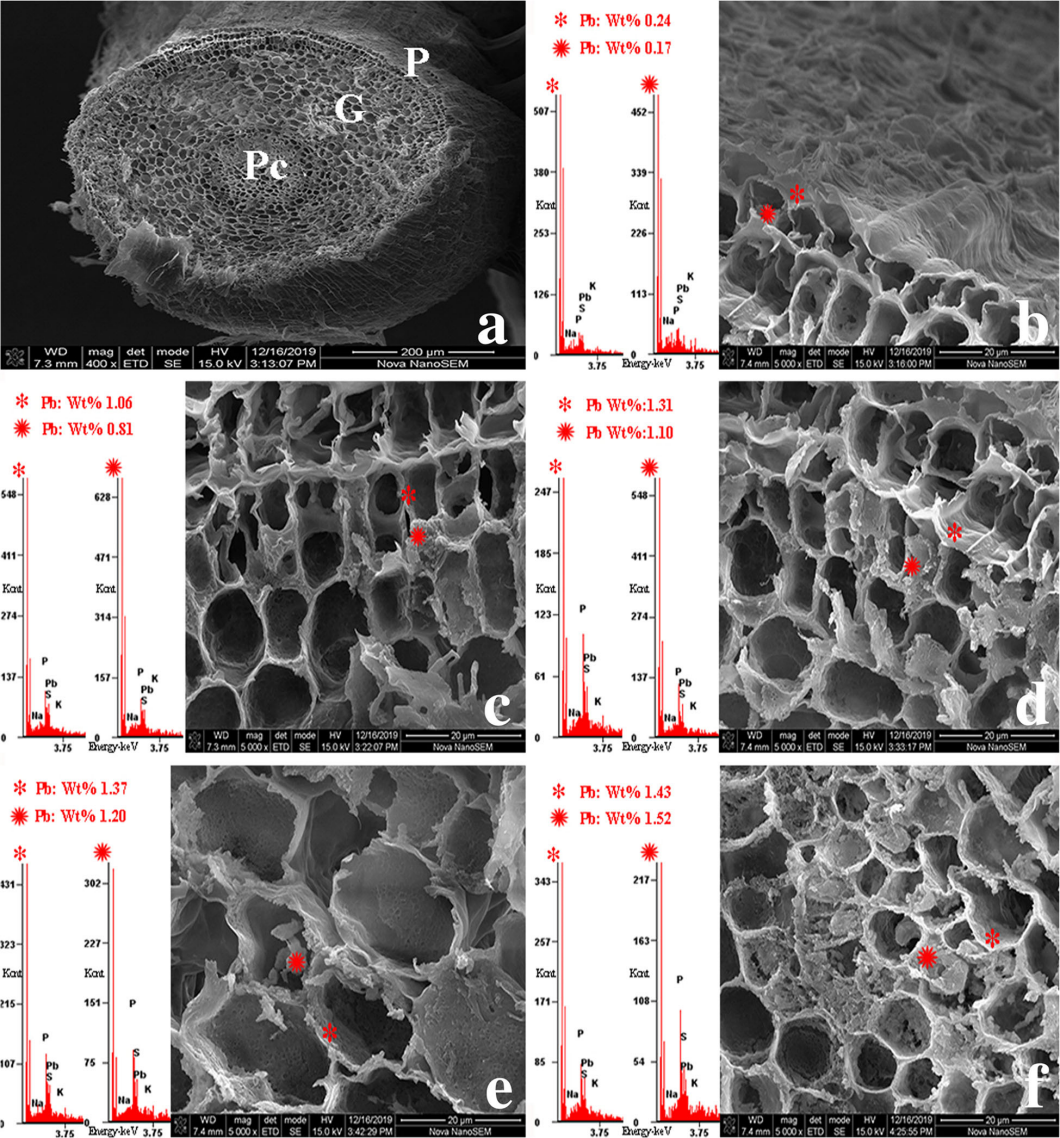
SEM micrographs and Pb localization at transverse sections in root meristem zone cells exposed to 50 μmol/L Pb for 24 h. **a**: Transverse section of meristem zone (scale bar = 200 μm), **b**: Protoderm (scale bar = 20 μm), **c–e**: Ground meristem (scale bar = 20 μm), **f**: Procambium (scale bar = 20 μm). *cell wall, cytoplasm, P: protoderm, G: ground meristem, and Pc: procambium

The SEM and EXDA results revealed the cellular localization of Pb in *S. babylonica* root tip cells exposed to 50 μmol/L Pb for 24 h, as well as the wt% of Pb localization in specific sites. At longitudinal sections, Pb ions were observed in the meristem, elongation, and mature zones of *S. babylonica* root caps exposed to Pb. Pb levels were ordered as follows: meristem zone (2.38 wt%) > elongation zone (1.37 wt%) > mature zone (1.10 wt%) > root cap (1.05 wt%) (Fig. 10). These findings exhibited the same trend as the Pb-specific Leadmium Green AM dye probe observations. In transverse sections of the mature zone, the epidermis, cortex, and vascular cylinder were easily distinguishable (Fig. 11a). The EDXA spectra revealed that Pb was located in the epidermis, cortex, and vascular cylinder after Pb stress. Pb distribution in these tissue cells was detected in both the cytoplasm and cell wall. Pb levels in the cell wall and cytoplasm of epidermal cells was the lowest compared to cortical and vessel cells. Pb content was ordered as follows: epidermal cells < cortical cells < vessel cells (Fig. 11b–f). Notably, an increasing trend in Pb content was detected in cortical cells from the epidermis to vascular cylinder (Fig. 11c–e). Pb levels were ordered as follows: cortical cells near vascular bundle > cortical cells between the epidermis and vascular cylinder > cortical cells near the epidermis (Fig. 11c–e). Pb levels in the protoderm, ground meristem and procambium of the transverse section of meristem were almost the same as those of the apical meristem (Fig. 12a). The Pb content in the cell wall and cytoplasm was ordered as follows: procambium > ground meristem > protoderm (Fig. 12b–f). The Pb content in the cell wall and cytoplasm of ground meristem gradually increased from the protoderm to procambium (Fig. 12c–e). Pb levels were ordered as follows: ground meristem cells near the protoderm < ground meristem cells from the protoderm to procambium < ground meristem cells near the procambium. These results demonstrated that the cell wall was the main Pb storage site in the ground meristem and protoderm. Moreover, the Pb levels in the procambium cytoplasm were higher compared to the ground meristem and protoderm. Based on the transverse sections, the Pb levels in mature zones were lower compared to the meristem zone.

## Discussion

According to Buscaroli [33], the transport of Pb from the roots to shoots is a critical step in Pb phytoextraction. The most recognized standard criteria are based on BCF (Bio-concentration factor) or TF as an indication of phytoremediation potential for different plant species. The plants exhibiting BCF or TF values ≥1 are considered as hyperaccumulators which are potential candidates for phytoextraction, and plants with the values < 1 are constituted metal excluders which are not suitable for phytoextraction [11, 33]. Notably, the TF value > 1 of Pb from *S. babylonica* roots to shoots is observed in all treatment groups throughout the experiment, so *S. babylonica* can be considered as a plant with great phytoextraction potentials. The data here show that *S. babylonica* has the ability to uptake and accumulate Pb. TF value > 1, that is to say, large amounts of Pb are transported to the shoots from the roots, which differs from other plants, including *Allium sativum*, *Ricinus communis*, *Brassica juncea*, *Neyraudia reynaudiana*, and some other willow clones, in which, large amounts of Pb ions are accumulated in the roots and small amounts are transported to the shoots after Pb stress [13, 14, 25, 34, 35]. Nevertheless, some studies have reported on the use of crop plants and forest plants, including poplar and willows, to remove heavy metals from contaminated soils [22, 23, 26, 36–40]. Other investigations indicated that hyperaccumulators, plants that accumulate heavy metals from the soil into their shoots, are immensely useful in phytoextraction [23, 36]. In this study, *S. babylonica* is used to evaluate its capability for TF > 1, demonstrating that *S. babylonica* is considered as a plant with great potential of Pb-accumulation. These results are in accordance with the findings of Chandrasekhar and Ray [11]. Pb accumulation in *S. babylonica* here is much lower than the three plants (*Eclipta prostrata* (L.) L., *Scoparia dulcis* L. and *Phyllanthus niruri* L.) reported by Chandrasekhar and Ray [41], which maybe due to the different Pb treatment method, Pb concentration and treatment time. Wang et al. [42] indicated that the transport from underground to above ground could be explained by the plants can pre-adapt and improve their tolerance to heavy metals through accumulating heavy metals in initial cuttings before rooted. Besides, more investigations are needed to carry out for further confirming the ability of *S. babylonica* in phytoremediation of metal contamination.

Roots contact lead and other heavy metals directly in soil system, which are sensitive to environmental stress. Root apical meristem is crucial in immediate stress response through the activation of signal cascades in other plant organs [15]. Excessive Pb often results in environmental contamination and inhibited plant growth. Therefore, understanding Pb uptake and accumulation in root sites, as well as evaluating the action mechanisms of Pb toxicity in plant root tip cells and their consequences on root growth and cell damage are very important. In this study, after a short exposure period, the results demonstrated that compared with the control Pb can restrain the growth of *S. babylonica* root, and the inhibition increased when Pb concentration increased, which is in accordance with the results reported by Jiang et al. [15], Khan et al. [18], Liu et al. [34], Wierzbicka [43], and Jiang and Liu [44]. However, further research is needed to assess the long-term ecological risks of Pb contamination under field conditions.

Fluorescent Pb reagents are rarely used in plant studies, however, Leadmium Green AM dye has been successfully used to detect Pb in plant roots [15]. The uptake of Pb in *S. babylonica* root cells was investigated using the Pb-sensitive Leadmium™ Green AM dye in this study. Green fluorescence, which represents the binding of the dye to Pb, was observed in the meristem zone of *S. babylonica* under Pb stress. An early study indicated that the meristem of plant roots is one of the most sensitive sites to Pb toxicity [43]. The results of this study demonstrated that Pb ions were first accumulated in the elongation zone of root tips after exposure to Pb and were gradually transported to the meristem zone after prolonged exposure, suggesting that the meristem of plant root tips is a target of Pb. In *S. babylonica* roots under Pb stress, the Leadmium Green AM data are very similar to the PI staining data, which demonstrates the existence of significant relevance between Pb accumulation and cell death in roots exposed to Pb. The results here supported previous observations in which Pb was absorbed within hours in *A. cepa* root cells exposed to Pb [15, 43]. Rucińska-Sobkowiak et al. [45] demonstrated that accumulated Pb caused enlargements of the apical meristems adjacent to root caps, leading to cell wall thickening and increased in the vacuole, which explained plant tolerance to Pb stress.

PI is an intercalating agent and fluorescent molecule used to stain DNA for studying cell membrane damage in plant roots after the exposure to heavy metals. The damage extent of cell membrane and morphological changes of cell membrane integrity can be reflected by the quantity of PI entered the cells [46–50]. In this study, the toxic effects of Pb on the cell membrane damaged *S. babylonica* root tip cells, which was confirmed by PI staining. The observed cell damage was mainly in the meristem and elongation zones of root tips exposed to 100 μmol/L Pb for 3 h (Fig. 4). The PI staining data are in agreement with the Pb absorption observations in different zones of *S. babylonica* roots under Pb stress. Under heavy metal stress, reactive oxygen species (ROS) production also increased. ROS interact with various cellular components and lead to oxidative damage in nucleic acids, proteins, sugars, and lipids, which in turn cause oxidative stress to the intracellular membrane [50]. Under Pb stress, ROS-induced oxidative stress leads to lipid peroxidation in cell membranes, which in turn produce malondialdehyde (MDA) [17, 51]. Cell damage in the roots exposed to Pb may be explained by the fact that Pb causes the lipid peroxidation of membranes and oxidative damage, leading to permeability and fluidity changes of the membrane lipid bilayer and altering cell integrity. Consequently, ROS-induced cellular damage induces local programmed cell death, which generally affects plant growth and development.

EDXA is an analytical technique used for analyzing the localization of elements in biological specimens at the subcellular level [48, 52]. In this study, EDXA at longitudinal sections showed that Pb ions accumulated in the meristem, elongation, and mature zones of *S. babylonica* root tips exposed to Pb, and the accumulation and distribution of Pb exhibited the same trend as the fluorescent probe results. Additionally, the cell wall is considered the primary Pb accumulation sites then the cytoplasm. Cell wall is the first barrier for heavy metal to enter cells. Plant reduces the toxicity of Pb by binding it to the cell wall, which is one of the mechanisms of plant tolerance. At transverse sections, Pb levels in the meristem zone were high compared to the mature zone, supporting the findings of Eun et al. [53], which demonstrated that Pb accumulation occurred in both the apoplast and symplast, and that the Pb levels in the root meristem were the highest. Root hairs are located only in the root mature zone and increase the absorption surface area greatly, making the uptake of water and minerals more efficiently during osmosis. After Pb ions enter the roots, they penetrate cortical tissues and are translocated to aboveground tissues. This explains why the Pb levels in the mature zone are low compared to the meristem zone. Moreover, the results demonstrated that the Pb levels were ordered as follows: epidermal cells < cortical cells < vessel cells (Fig. 11b–f), which determined that Pb was easily translocated from roots to the aboveground part through vascular tissue. The Pb levels in the meristem zone were higher compared to the mature zone, indicating that the *S. babylonica* root tip meristem is a target of Pb accumulation and toxicity. Meristem cells are small and have thin walls without differentiation. Because these cells have not yet differentiated, they have a poor transport capacity. Thus, excess Pb can easily damage cell construction, thereby inducing a low mitotic index and production of large damaged cells that inhibit *S. babylonica* seedlings.

## Conclusions

Based on the results obtained in this investigation, we can draw the following conclusions. Under Pb stress, Pb ions initially entered elongation zone cells and gradually accumulated in the meristem zone, and they were localized primarily to the cell wall then to cytoplasm. Pb level in epidermal cells was the lowest compared to cortical and vessel cells, and there was an increasing trend in cortical cells from the epidermis to vascular cylinder. Cell damage in the roots exposed to Pb detected by PI staining was in agreement with the findings of Pb absorption in different zones of *S. babylonica* roots under Pb stress by SEM with EDXA. At 100 μmol/L Pb treatment for 7 d, the root Pb accumulation was 78.78 ± 0.34 μg/g dry weight of the tissue, and the shoot Pb accumulation was 151.37 ± 0.16 μg/g dry weight of the tissue. The TF value showed > 1 in all treatment groups, although Pb exerted inhibitory effects on the root and shoot growth. Based on these characteristics, *S. babylonica* could be thought to have great potential for phytoextraction to Pb after the short-term investigation. The information obtained here would lead to a better understanding of Pb resistance and tolerance mechanisms, which would provide valuable and scientific information for phytoremediation investigations of other woody plants stressed by Pb. However, further investigation on long-term Pb accumulation and distribution at even higher concentrations of Pb treatment is still required.

## Methods

### Plant materials and growth conditions

*S. babylonica* used in this experiment was identified and offered by Professor Wenhui Zhang of Northwest A&F University, China. The collection of the experimental materials conforms to the institutional, national or international guidelines. Healthy woody cuttings (25 cm long) from 1-year-old *S. babylonica* shoots grown on the campus of Tianjin Normal University, China were collected and rooted in plastic buckets that contained distilled water. Seven-day-old woody cuttings with new roots were transferred to half-strength Hoagland nutrient solution containing 0, 1, 10, 50, or 100 μmol/L Pb and grown for 7 d. The nutrient solution consisted of 5 mM Ca (NO_3_)_2_, 5 mM KNO_3_, 1 mM KH_2_PO_4_, 1 mM MgSO_4_, 50 μM H_3_BO_3_, 10 μM FeEDTA, 4.5 μM MnCl_2_, 3.8 μM ZnSO_4_, 0.3 μM CuSO_4_, and 0.1 μM (NH_4_)_6_Mo_7_O_24_ adjusted to pH 5.5. Control seedlings were grown in the nutrient solution alone. Solutions were continuously aerated with an aquarium air pump. Experiments were conducted in a greenhouse under a 14/10 h light/dark photoperiod at 26/18 °C (day/night) and 65–75% humidity. The roots were protected from direct sunlight. Pb was supplied as lead nitrate [Pb (NO_3_)_2_]. All treatments were performed in triplicate. TF were calculated as follows [41]:

$$ \mathrm{TF}=\frac{\mathrm{Metal}\kern0.5em \mathrm{concentration}\kern0.5em \mathrm{in}\kern0.5em \mathrm{shoot}}{\mathrm{Metal}\kern0.5em \mathrm{concentration}\kern0.5em \mathrm{in}\kern0.5em \mathrm{root}}\kern0.5em $$

### Determination of Pb

Control and experimental plants stressed by 0, 1, 10, 50, or 100 μmol/L Pb for 7 d were harvested randomly. In order to get rid of the traces of nutrients and Pb ions on their surfaces, the root samples were washed with running tap water for 30 min, 20 mM disodium ethylenediamine tetraacetic acid (Na_2_-EDTA) for 10 min and deionized water for 3 min in turn. Plant tissues were divided into roots and shoots (i.e., leaves, new stems, and old stems). Roots were dried at 45 °C for 72 h, 80 °C for 24 h, and 105 °C for 12 h, then ground with a cutting mill (IKA-Werke GmbH & CO. KG, Staufen, Germany). After weighing, dried-root material (0.2 g) was digested with a mixture of HNO_3_ and HClO_4_ (4:1, v/v) at 160 °C. Dried plant samples were prepared using the wet-digestion method [34]. After dry-ashing, Pb concentrations were analyzed using ICP-AES (Leeman Labs Inc., Hudson, NH, USA).

### PI staining

*S. babylonica* root tips exposed to different Pb concentrations (0, 1, 10, 50, or 100 μmol/L) for 3, 6, 12, and 24 h were washed three times with phosphate-buffered saline (PBS, pH 7.0). Samples were soaked in 1 mmol/L PI (Sigma-Aldrich, Buchs, Switzerland) at 25 °C for 8 min in the dark, and then thoroughly washed with phosphate buffer (50 mmol/L, pH 7.8). According to the methods of Zou et al. [54], Eclipse 90i laser confocal scanning microscope (Nikon Corp., Tokyo, Japan) was adopted to examine the samples, with the excitation maximum set at 535 nm and that of fluorescence emission at 617 nm. Due to the extremely low penetrability across intact membranes, red fluorescence triggered by PI can only be observed in the nuclei of damaged cells. Red fluorescence is an indicator of cell damage [50, 54]. Fluorescence density was analyzed using the “Analyze and Measure” function in Image J software (NIH, Bethesda, MD, USA).

### Fluorescence labelling of Pb

*S. babylonica* root tips exposed to different Pb concentrations (0, 1, 10, 50, or 100 μmol/L) for 3, 6, 12, and 24 h were soaked in EDTA solution (Na_2_-EDTA, 20 mmol/L) and washed with running water for 15 min. Then, root tips were washed with deionized water 3 times. Afterwards, experimental and control roots were stained using the Pb-specific probe Leadmium™ Green AM solution (Molecular Probes, Invitrogen, Carlsbad, CA, USA) for 90 min at 40 °C in the dark following the manufacturer’s instructions to visualize Pb absorption and distribution [55]. Intact cells exhibited green fluorescence due to the Pb-specific probe Leadmium™ Green AM solution. Fluorescence density was analyzed using the “Analyze and Measure” function in Image J software to evaluate the Pb distribution in intact roots. Prepared samples were observed using a Nikon Eclipse 90i confocal laser scanning microscope with an exciter at 488 nm and a barrier at 590/50 nm.

### Sample preparation for SEM and EDXA

The elemental distribution and composition of experimental plants were determined from freeze-dried root materials. *S. babylonica* roots treated with 50 μmol/L Pb for 24 h were removed from the Pb (NO_3_)_2_ solution and washed thoroughly. Samples (1 cm long) were cut from the root tips, soaked in 20 mM EDTA-NO_2_ solution for 15 min, and washed three times with ddH_2_O for 10 min. Materials were washed three times with PBS (pH 7.2) for 10 min. According to the methods referred by Shi et al. [52], the root tips were frozen quickly in liquid nitrogen and lyophilized in vacuum. An Emitech K550X sputter/coater (Quorum Group, London, England) was adopted to gild the root samples. A FEI Nova NanoSEM 230 (FEI Company, Oregon, USA) with a Genesis Apollo 10 EDXA (FEI Company, Oregon, USA) was employed to carry out EDXA. Via an X-ray and X-ray detector with a super ultra-thin window, the spectra were collected at 20 kV for 30–40 s. Pb contents were calculated as weight percent (Wt%) (i.e., the weight-based (or mass) percent concentration of a certain element relative to the gross element weight (or mass)).

### Statistical analyses

Fifteen seedlings were involved in each treatment, which was repeated five times to achieve statistical validity. SPSS v17.0 (SPSS Inc., Illinois, USA) and SigmaPlot v8.0 (Systat Software Inc., San Jose, CA) were adopted to analyze the results. The data are expressed as the mean ± standard error (SE). A one-way analysis of variance (ANOVA) was applied to determine the differences between treatments. In the case of *p* < 0.05, the results were deemed as statistically significant.

## Data Availability

The datasets used and/or analysed during the current study are available from the corresponding author on reasonable request.

## Abbreviations

Pb: Lead
ICP-AES: Inductively coupled plasma atomic emission spectrometry
TF: Translocation factor
BCF: Bio-concentration factor
PI: Propidium iodide
SEM: Scanning electron microscopy
EDXA: Energy-dispersive X-ray analyses
ROS: Reactive oxygen species
MDA: Malondialdehyde
PBS: Phosphate buffer saline
SE: Standard error
ANOVA: Analysis of variance
